# Metagenomic Profiling of Oral Microbiome Dynamics During Chemoradiotherapy in Head and Neck Squamous Cell Carcinoma Patients

**DOI:** 10.1002/cam4.70589

**Published:** 2025-01-13

**Authors:** Dominique A. Torozan, Cédric Christian Laczny, Kirsten Roomp, Paul Wilmes, Jochen Fleckenstein, Jochen G. Schneider

**Affiliations:** ^1^ Department of Radiotherapy and Radiation Oncology, University Hospital Saarland University Homburg Germany; ^2^ Luxembourg Centre for Systems Biomedicine University of Luxembourg Belvaux Luxembourg; ^3^ Department of Radiotherapy Westpfalz‐Klinikum Kaiserslautern Germany; ^4^ Department of Internal Medicine II, Gastroenterology, Hepatology, Endocrinology and Diabetology, University Hospital Saarland University Homburg Germany; ^5^ Department of Life Sciences and Medicine (DLSM) University of Luxembourg Belvaux Luxembourg

**Keywords:** chemoradiotherapy, head and neck cancer, metagenomic sequencing, oral microbiome, radiation‐induced oral mucositis

## Abstract

**Background:**

We explored the interaction between the oral microbiome and the development of radiation‐induced mucositis in patients with head and neck squamous cell cancer (HNSCC) undergoing chemoradiotherapy (CRT). We prospectively studied the oral microbiome and compared it to healthy controls. Additionally, we compared patients with low‐grade (LGM) vs. high‐grade mucositis (HGM).

**Methods:**

Ten HNSCC patients scheduled for CRT were included. Saliva samples were characterized prior to, during, and nine months after CRT using metagenomic sequencing. We similarly characterized samples from seven healthy controls. We assessed alpha and beta diversity and examined abundances at different taxonomic levels between (sub)groups.

**Results:**

Patients exhibited significantly reduced alpha diversity compared to controls at all times (p ⟨ 0.05). Differential abundance of taxa between patients and controls was observed at baseline. In patients, the relative abundance of *Staphylococcus aureus* and *Escherichia coli* increased significantly during CRT. *Capnocytophaga* spp. was associated with the definitive CRT patients' subgroup. At baseline, two fungal families (Melampsoraceae and Herpotrichiellaceaea) were more abundant in patients who later developed HGM. No differentially abundant taxa were found between LGM vs. HGM during irradiation.

**Conclusion:**

Our findings support the hypothesis that CRT, as well as HNSCC itself, influences the composition of the oral microbiome. Microbial markers found in patients who later developed HGM should be evaluated using independent cohorts to qualify their specific biomarker potential.

## 1 Introduction

Head and neck squamous cell carcinoma (HNSCC) represents a heterogeneous group of malignant neoplasms and belongs to the 10 most common tumor entities worldwide. In addition to alcohol and tobacco consumption, chronic HPV infections are a common risk factor for the development of oropharyngeal squamous cell carcinomas (OPSCCs). Treatment for HNSCCs aims to eradicate tumor cells while minimizing both acute and late toxicities and includes local methods, such as surgery, chemoradiotherapy (CRT), and systemic approaches, such as immunotherapy. When appropriate, intensity‐modulated radiation therapy with concomitant cisplatin‐based chemotherapy represents the gold standard. As HPV‐related OPSCC is extremely sensitive to radiation exposure, patients with locally advanced HPV‐positive oropharyngeal cancer have improved outcomes relative to patients with HPV‐negative tumors [1].

Virtually every patient with HNSCC undergoing CRT experiences radiation‐induced inflammation of the oral and pharyngeal mucosa, typically referred to as radiation‐induced oral mucositis (RIOM) in the irradiated area of varying clinical severity [2]. RIOM is not only associated with severe pain, dysphagia, dysgeusia, and xerostomia but can also lead to interruption of CRT, prolonged hospitalization, and an increased risk of local and systemic infection [2]. Treatment deintensification strategies, such as dose‐reduced CRT, are an active area of research with promising preliminary results in HPV‐related OPSCC, especially because HPV positivity was established as a favorable prognostic factor [3].

The pathogenesis of RIOM is not fully understood, and there are few effective prevention strategies [4]. Genetic factors, such as drug metabolism, TNF‐alpha gene polymorphism, or polymorphism of genes responsible for protection against reactive oxygen species (ROS) affect the severity of RIOM [5] but can only partially explain why RIOM severity can vary substantially among individuals at identical dose levels.

The role of microorganisms in the pathogenesis and progression of RIOM is thus a subject of current research efforts. It is of interest whether the oral microbiota contributes to the pathogenesis of RIOM [4] alongside treatment‐related and patient‐related risk factors or is a consequence of it [6]. As to size and diversity, the microbiome of the oral cavity ranks second after the gut microbiome. It includes not only bacteria and viruses but also archaea and fungi and harbors over 700 microbial species, of which 30% remain uncultivated under laboratory settings [7]. The human microbiome has a low level of pathogenicity, but certain species of the homeostatic microbiota or exogenous pathogens may become opportunistic pathogens in cases of tissue injury or lack of adequate oral hygiene, or due to immune deficiency [8].

Using both culture‐dependent and culture‐independent methods, it has been shown that CRT compromises oral defense mechanisms and causes distinct shifts in the oral microbiota [9]. Direct cytotoxic effects of radiation and chemotherapeutic drugs, but also radiotherapy‐caused hyposalivation, may affect the oral microbiota [10].

The rapid development in high‐throughput sequencing technology (“next generation sequencing” [NGS]) and its application to the study of the entire genomic complement of microbiomes, also called metagenomic sequencing, drastically improved our ability to understand the role of the oral microbiome in health and disease [11]. Nevertheless, there is no study to our knowledge investigating the shifts of the oral microbiome in patients with HNSCC during CRT using metagenomic sequencing. Furthermore, only sparse data exist on the role of the oral microbiome or consistent correlations between the presence of specific pathogens and the onset, severity, and duration of RIOM [12]. Therefore, we performed metagenomic sequencing to study potential changes in the oral microbiome of HNSCC patients undergoing CRT over time and compared these changes to a healthy control group. Furthermore, we investigated a possible correlation between the severity of RIOM and the dynamics of the oral microbiome.

## 2 Materials and Methods

The study design was approved by the local ethics committee. A written informed consent form (ICF) was signed by each participant before inclusion. In our prospective longitudinal study, saliva samples were taken from HNSCC patients (*n* = 10) before the commencement of CRT, during CRT, and 9 months after completed tumor therapy to investigate potential influences of CRT on the oral microbiota profiles (Table S1).

To assess the influence of CRT regarding potential changes in the patient's oral microbiome and to minimize potential confounding by natural microbial shifts over time, we additionally monitored saliva samples from healthy individuals (*n* = 7) over a period of 3 months. In total, we collected 58 samples from March 2017 to March 2019. The baseline characteristics are summarized in Table 1.

**TABLE 1:** Baseline characteristics, tumor histology, tumor stage (UICC eighth version p16 positive oropharyngeal carcinoma), and therapy regimen.

Variables	Patients (*n* = 10)	Controls (*n* = 7)	*p*
Age	64.4 ± 7.7	31 ± 9.5	0.001
Male	10 (100%)	2 (28.6%)	0.001
BMI	28 ± 6.5	24.73 ± 5.4	0.204
Aktive smoking	1 (10%)	3 (42.9%)	0.116
Ex‐smoking	7 (70%)	0	0.004
Pack years	41 ± 34	8 ± 2	0.195
Karnofsky performance status scale (%)	90 (80–90)	100 (100–100)	< 0.0001
Never smoked	2 (20%)	4 (57.1%)	0.155
Head and neck squamous‐cell carcinoma (HNSCC)	10 (100%)	n/a	
T 3–4	4 (40%)	n/a	
N+	8 (80%)	n/a	
M0	10 (100%)	n/a	
HPV+	5 (50%)	n/a	
HPV−	5 (50%)	n/a	
Oral cavity contoured volume (mL)	92.34 ± 11.62	n/a	
Oral cavity mean dose (Gy)	62.64 ± 5.67	n/a	

Abbreviations: BMI = body mass index, HPV = human papilloma virus, N/a = not applicable.

All patients received radiotherapy for their HNSCC according to the standard of care over 6 weeks in combination with platinum‐based chemotherapy (nine cisplatin, one cisplatin + paclitaxel according to PACCIS regimen). Oropharyngeal carcinoma was present in eight patients; the remaining two patients had floor of mouth carcinoma (Figure S2). All patients were treated with a complex intensity‐modulated radiotherapy (IMRT) technique. Dose constraints for the parotid and submandibular glands were applied according to QUANTEC criteria. However, dose constraints could not be met for the ipsilateral parotid and submandibular glands in 7 out of 10 patients due to the close proximity of the target volume. Criteria for the contralateral glands were met in all patients. The delineation and selection of lymph node stations were performed according to an internal standard operating procedure, which was based on the publication by Grégoire et al. [13].

The median reference dose was 66 Gray with a range from 59.92 to 72 Gray. All patients were able to complete radiotherapy. Two out of 10 patients (Patients 6 and 7) were not able to donate saliva samples in Week 6 of CRT due to xerostomia, so we used their samples from Week 5 for our analyses instead. Nine months post‐therapeutically, we could not collect saliva samples from Patients 1, 3, and 9 due to xerostomia. Four patients underwent additional antibiotic therapy (ABT) along with CRT, which was highly individual and adapted according to clinical requirements as shown in Table 2.

**TABLE 2:** Patient therapies.

Patient	Age	Smoking status	Cancer site	Therapy	CRT regime	ABT	Mucositis (CTCAE/WHO)
1	69	Ex‐smoking	Oropharynx	aCRT	66 Gy à 2 Gy/1× daily, 5× weekly 6× cisplatin (weekly)		Low grade
3	69	Never smoked	Oropharynx	aCRT	59.92 Gy/1× daily, 5× weekly 6× cisplatin (weekly)	F	Low grade
4	67	Ex‐smoking	Oropharynx	dCRT	63.6 Gy/1× daily, 5× weekly 2× Cisplatin + Paclitaxel (week 1 + 4)	C, U	High grade
5	68	Active smoking	Oral cavity	aCRT	66 Gy à 2 Gy/1× daily, 5× weekly 6× Cisplatin (weekly)	F, C, V	Low grade
6	50	Ex‐smoking	Oropharynx	aCRT	59.92 Gy/1× daily, 5× weekly 6× Cisplatin (weekly)		Low grade
7	70	Ex‐smoking	Oropharynx	aCRT	60 Gy à 2 Gy + 1 LN 67.5 Gy/1× daily, 5× weekly 6× Cisplatin (weekly)		High grade
8	65	Ex‐smoking	Oropharynx	dCRT	72 Gy/2× daily, 5× weekly 6× Cisplatin (weekly)		High grade
9	69	Ex‐smoking	Oral cavity	dCRT	72 Gy/1× daily, 5× weekly 6× Cisplatin (weekly)^a^	D^b^	High grade
11	78	Never smoked	Oropharynx	dCRT	70 Gy/1× daily, 5× weekly 6× Cisplatin (weekly)	F	High grade
12	72	Ex‐smoking	Oropharynx	dCRT	70 Gy/1× daily, 5× weekly 6× Cisplatin (weekly)		None

Abbreviations: ABT = antibiotic therapy, C = clindamycin, D = doxycycline, F = fluoroquinolones, U = unacid, V = vancomycin.

^a^ Sixth cycle not administered (infection), aCRT = adjuvant chemoradiotherapy, dCRT = definitive chemoradiotherapy.

^b^ During follow‐up.

If necessary, dental restoration was carried out in patients before the start of CRT. In addition, a standardized mouthwash protocol was used throughout the entire CRT to optimize oral hygiene and to simplify comparisons between patients. Ideally, a saliva volume of 3–5 mL was to be collected in a provided test tube. A minimum saliva volume of 2 mL was accepted. The sample was immediately stored at −80°C until further use. The mucosa of the patient's oral cavity was inspected and scored clinically according to the World Health Organization (WHO) and Radiation Therapy Oncology Group (RTOG) criteria [14, 15]. Furthermore, besides a routine examination and clinical chemistry, oral hygiene, nicotine, and alcohol consumption were monitored.

After saliva sample collection was completed, the material was sent to Novogene (Cambridge, UK) for DNA extraction and for metagenomic sequencing. The microbial DNA was isolated and purified according to the protocol of the Magnetic Swab DNA Kit as specified by the manufacturer (Tiangen, 4992410/4992411, RRID:SCR_023688). After quality control, a total amount of 1 μg DNA per sample was used to create sequencing libraries. The sequencing libraries were prepared using the NEBNext Ultra DNA Library Prep Kit for Illumina (NEB, USA) according to the manufacturer's recommendations. The nucleic acids were labeled with index codes to assign sequences to each sample.

In the first step, the genomic DNA was mechanically fragmented to a size of 350 base pairs (bp). The resulting fragments were end repaired, polyadenylated, and ligated with Illumina adapters. The ligated fragments were further processed by Illumina paired‐end sequencing and amplified by PCR. In the next step, the PCR products were purified (AMPure XP System) and selected by size (Agilent 2100 Bioanalyzer Instrument, RRID:SCR_018043). This was followed by amplification of the selected and purified DNA fragments using real‐time PCR. The amplified clusters were then sequenced on an Illumina HiSeq platform (Illumina Inc., CA, USA, RRID:SCR_010233).

After sequencing, the metagenome of each sample was assembled using both reference‐based and de novo assembly. Reads that were not used in the initial assembly were pooled together for mixed assembly to explore the information on low‐abundance species in the samples. Gene prediction was done using MetaGeneMark, a gene catalog was created, and abundance information was obtained. In the next step, the metagenomic reads were compared with the database of taxonomically informative gene families and assigned to the respective microbial taxonomy. In addition to the taxonomic analysis, according to the database for antibiotic resistance genes (CARD), the frequency of antibiotic resistance genes (ARGs) was determined [16]. Raw sequencing files obtained in this study can be made available on request.

Alpha diversity was calculated using two common alpha diversity indices, the Shannon index and the Chao 1 index, at the genus level [17, 18]. Differences in beta diversity were computed using analysis of similarity (ANOSIM) [19]. To further identify which microbial taxa were distinct between the groups, the linear discriminant analysis effect size (LEfSe) method [20] and multivariate statistical analysis of Metastats [21] were applied. The visualization of the results from LEfSe analyses and Metastats analyses was done using box‐plot diagrams and heat maps. To visualize microbial dissimilarity, principal component analysis (PCA), principal coordinates analysis (PCoA), and nonmetric multi‐dimensional scaling analysis (NMDS) were used [22]. We assessed differences in overall microbial community composition by using Bray–Curtis dissimilarity [23]. Bar charts were used to show relative taxonomic abundances on different taxonomic levels or relative percentages of ARGs.

## 3 Results

### 3.1 Comparison of the Oral Microbiota of HNSCC Patients and Healthy Individuals Before, During, and After CRT

**FIGURE 1:**
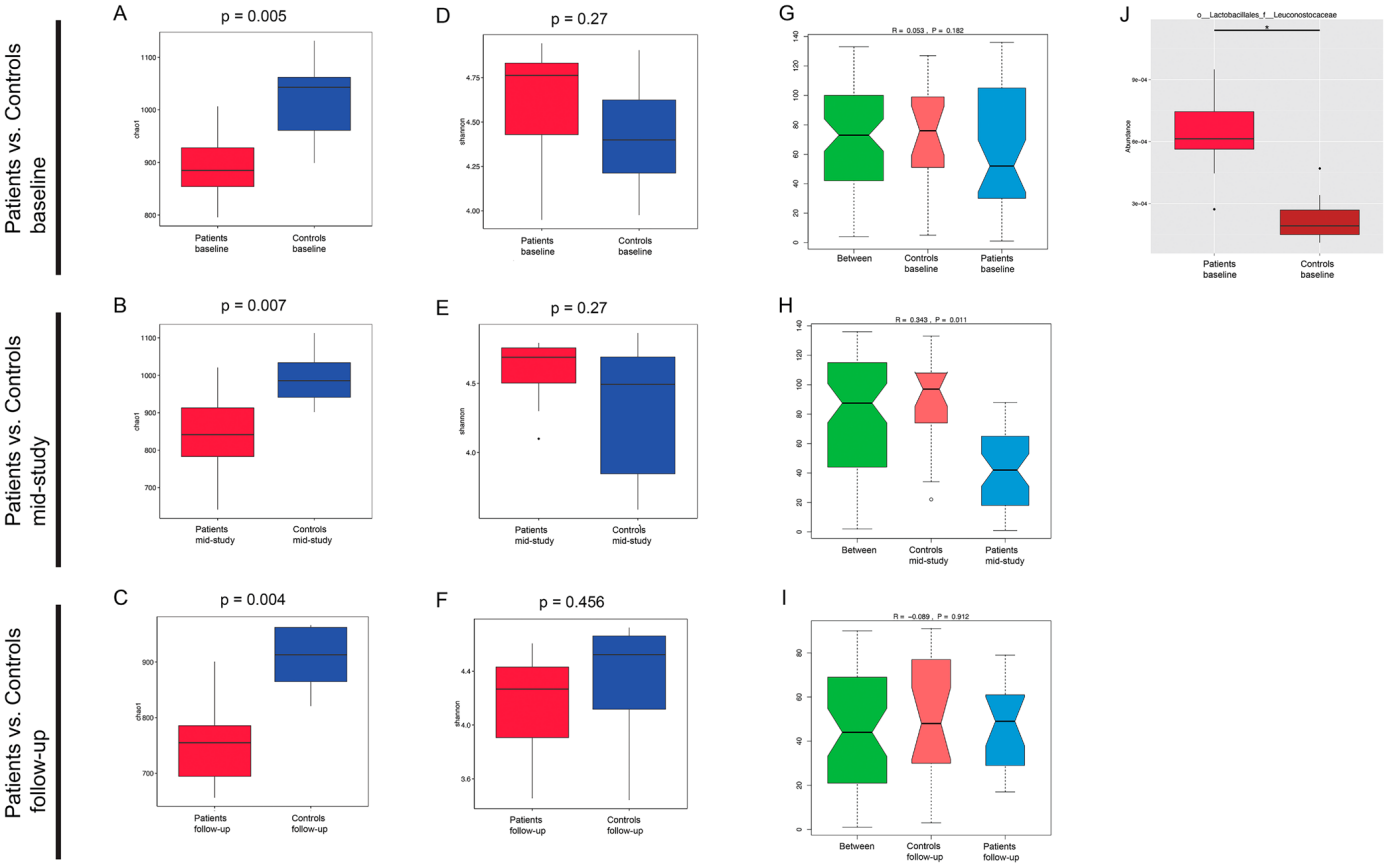
Evaluation of differences between patient and control samples. (A,B,C,D,E,F) Comparison of alpha‐diversity indices (Shannon and Chao1) of patient samples and control samples at different study time points. In all box plots: box hinges: 1st and 3rd quartiles; whiskers: hinge to highest/lowest values that are within 1.5*IQR of hinge. (G,H,I) ANOSIM analysis comparing patient and control samples at different time points with respect to their similarity at the phylum level. The green boxplot shows the inter‐group variance, while the red boxplot shows the intra‐group variance of the control cohort and the blue boxplot shows the intra‐group variance of the patient cohort. (J) Comparison of relative abundance of the Leuconostocaceae family taxon in patient samples and control samples at baseline. Significantly different relative abundance (“*” = q < 0.05) was found.

First, we compared the oral microbiome of HNSCC patients and healthy controls. Our results showed significantly lower Chao 1 index values in HNSCC patients as compared to controls (*p* < 0.05) at all time points (Figure 1A,B,C). The Shannon index values did not differ significantly between patients and controls (Figure 1D,E,F). We did not detect a significant difference in beta diversity when comparing patients and controls at baseline and during follow‐up (Figure 1G,I), but we found that the intergroup variance of HNSCC patients during CRT and controls at the phylum level was higher than the intragroup variance (*p* = 0.011) (Figure 1H).

Mid‐study, the salivary microbial community was dominated by the following five phyla in patients versus controls (Figure S1A): Firmicutes (9.29% vs. 19.81%), Actinobacteria (1.81% vs. 14.92%), Proteobacteria (9.85% vs. 6.72%), Bacteroidetes (2.94% vs. 3.66%), and Fusobacteria (0.55% vs. 0.87%). Low‐abundant sequences or sequences that could not be classified into any known groups were grouped as “others” (73.4% vs. 52.13%). On the genus level, the five most abundant taxa in patients versus controls were *Streptococcus* spp. (3.56% vs. 10.1%), *Actinomyces* spp. (0.15% vs. 5.24%), *Rothia* spp. (0.39% vs. 3.73%), *Neisseria* spp. (2.0% vs. 0.79%), *Veillonella* spp. (0.53% vs. 2.39%), and “others” (89.36% vs. 71.98%) (Figure S1B).

Twelve taxa with significantly higher relative abundance in the control group were found at baseline, such as from the Actinobacteria class, including the *Rothia* genus and *Rothia mucilaginosa* species, the *Candidatus* Saccharibacteria phylum, the Sanguibacteraceae family including the *Sanguibacter* genus and *Sanguibacter keddieii* species, as well as *Gemella sanguinis* species, among others (Figure S2A). Moreover, the Metastats analysis revealed the Leuconostocaceae family of the Lactobacillales order to be significantly increased in its relative abundance in patients at baseline (Figure 1J) compared to healthy controls.

**FIGURE 2:**
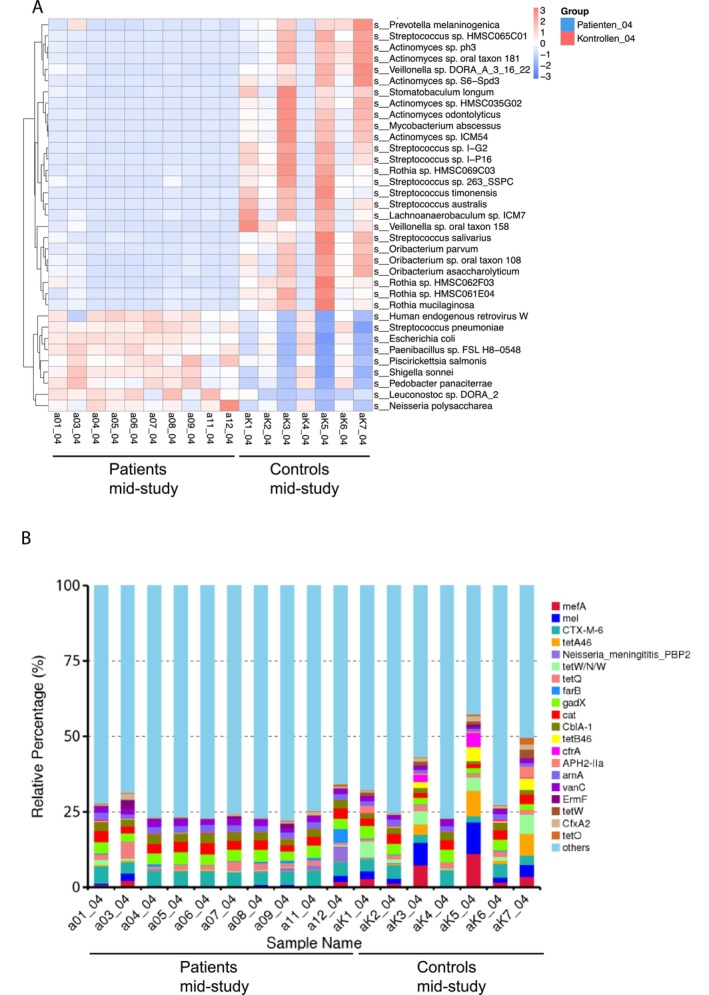
Comparison of significantly differentially abundant species and relative abundances of antimicrobial resistance (AMR) genes between patients and controls at mid‐study. (A) Relative abundances of 35 significantly differentially abundant species between patients and controls are shown. The color code ranges from blue (low relative abundance) to red (high relative abundance). The dendrogram on the left side of the heat map shows the clustering of species based on their relative abundance across samples. (B) AMR gene profiles of patient samples and control samples at mid‐study. The top 20 most abundant AMR genes in terms of relative abundance are shown. All other genes are grouped under “Others.”

During CRT (mid‐study), LEfSe analysis detected 36 taxa to be enriched in controls compared to patients (Figure S2B). All 12 taxa already found to be enriched in controls at baseline were also enriched in controls compared to patients during CRT. On the genus level, *Streptococcus* spp., *Rothia* spp., *Veillonella* spp., *Actinomyces* spp., *Atopobium* spp., and *Haemophilus* spp. had a significantly higher relative abundance in controls compared to patients mid‐study. Moreover, we found 35 microbial species with significant differences in relative abundance between patients during CRT (mid‐study; Figure 2A) and the control group. Nine of the 35 species were enriched in patients (*q* < 0.05), including, for example, *Human endogenous retrovirus W*, *Streptococcus pneumoniae* , and *Escherichia coli* , whereas 26 species were enriched in the control group, for example, different *Streptococcus* spp., such as *Streptococcus salivarius*, *Streptococcus timonensis* and *Streptococcus australis*, as well as various *Actinomyces* spp.

Six taxa differed significantly between patients and controls during follow‐up (Figure S2C). Unclassified Bacillales and *G. sanguinis* were enriched in controls during follow‐up, whereas the Neisseriales order, including *Neisseria* spp. and *Megasphaera micronuciformis* , was enriched in patients.

Following the analysis of differentially abundant taxa, we assessed the frequency of ARGs and their distribution (Figure 2B). ARGs with the highest relative percentages in the patients' group during CRT were CTX‐M‐6 (4.81%), gadX (3.58%), and cat (3.09%). In the control group, the ARGs with the highest relative percentages were mefA (4.02%), followed by mel (3.98%), CTX‐M‐6 (3.74%), tetW/N/W (3.32%) and gadX (3.03%). The relative percentages of 10 ARGs were higher in the patients' group during CRT compared to controls, namely, CTX‐M‐6, *Neisseria meningitidis* PBP2, tetQ, farB, gadX, cat, CblA‐1, arnA, vanC, and ErmF.

### 3.2 Characterization of Longitudinal Changes in the Oral Microbiome of the Patient Collective Before, During, and After CRT

To further study the potential impact of cumulative radiation dose and mucositis severity on patients' oral microbiota, we compared patients' saliva samples longitudinally before and during CRT. First, we formed subgroups according to treatment modality to identify possible differences in the salivary microbiome of patients treated with definitive chemoradiotherapy (dCRT, *n* = 5) versus adjuvant chemoradiotherapy (aCRT, *n* = 5) and analyzed them after tumor operation but before commencement of CRT at baseline. We could not find statistically significant differences in the alpha or beta diversity indices between dCRT and aCRT at baseline (Figure S3A–C). Interestingly, linear discriminant analysis (LDA) scores showed *Capnocytophaga* spp. to be relatively more abundant in the dCRT subgroup at baseline (Figure S4A).

Then, we compared all patient samples at baseline, mid‐study (CRT Week 3), and end of study (CRT Week 6). Nine out of 10 patients developed RIOM. RIOM normally started in CRT Week two and peaked around Weeks 5 to 6. During follow‐up, no patient suffered from RIOM.

The Chao 1 index on the genus level was significantly higher in patients at baseline versus patients at the end of study, whereas the Shannon index showed no significant differences. The beta diversity on the phylum level displayed a significant difference between patients at baseline versus patients at the end of study as the intergroup variance was higher than the intragroup variance (*p* = 0.008) (Figure S3F).

**FIGURE 3:**
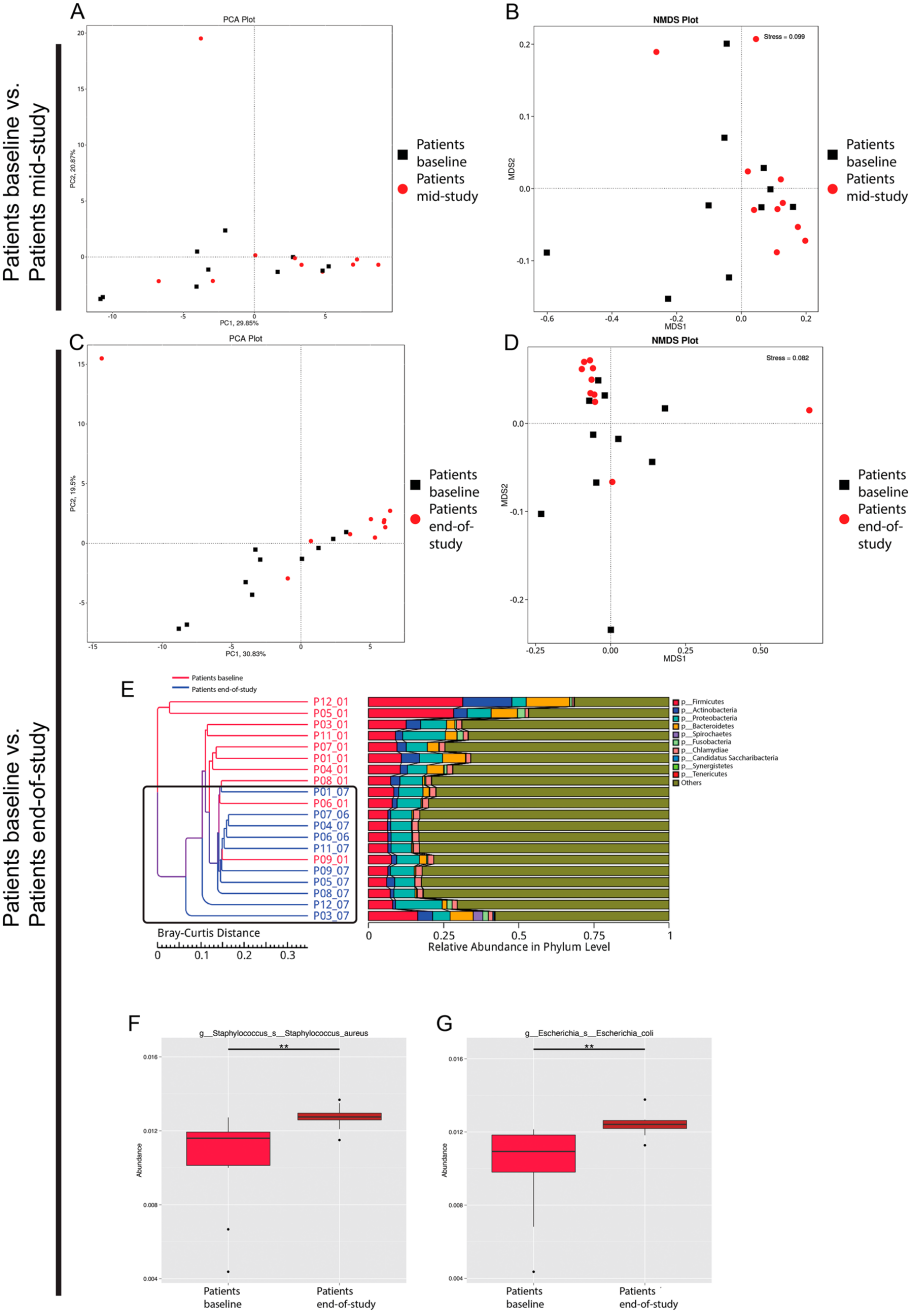
Evaluation of the differences in saliva microbiota of patients over time. (A, C) Principal component analysis (PCA) and (B, D) nonmetric multi‐dimensional scaling analysis (NMDS) representing the overall structure of patient samples' microbiota. (E) Sample clustering analysis based on Bray–Curtis distance of the relative taxonomic abundance at the phylum level. (F, G) Microbial taxa with significantly different relative abundances (“**” = *q* < 0.01) comparing patients at baseline versus at the end of study.

Our analyses revealed an overlap between patient samples at baseline and patient samples mid‐study (CRT Week 3) (Figure 3A,B), whereas the microbial communities clustered more closely together at the end of the study and became more distinguishable compared to samples at baseline (Figure 3C,D). Hierarchical clustering analysis based on Bray–Curtis distance showed that microbial profiles of patients at baseline were generally closer to each other than that of patients at the end of study (CRT Week 6) and vice versa (Figure 3E).

We found 24 taxa to be significantly more abundant in patients at baseline compared to patients at the end of study (Figure S4B) at the genus level, namely, *Streptococcus* spp., *Veillonella* spp., *Prevotella* spp., *Neisseria* spp., *Rothia* spp., *Haemophilus* spp., *Stomatobaculum* spp., and *Actinomyces* spp. Two species were found to be associated with patients at the end‐of‐study, namely, *S. aureus* and *E. coli* (Figure 3F,G).

To explore the potential impact of ABT on the oral microbiome during CRT, we analyzed samples from patients before and during ABT. No significant differences were found in the alpha and beta diversity of the microbial profiles of patients before and during the use of antibiotics (Figure S3G–I). Also, no taxa were found to have statistically significantly different relative abundances.

### 3.3 Comparison of the Oral Microbiota of Patients With Low‐Grade Mucositis Versus High‐Grade Mucositis

Next, we formed subgroups of patients' saliva samples according to mucositis severity (low‐grade mucositis [LGM] vs. high‐grade mucositis [HGM]) and compared them at different time points to gain additional insight into the relationship between the oral microbiome and the incidence of HGM. Five patients suffered from HGM, defined as mucositis ≥ grade 3 on the RTOG and/or WHO scale, whereas four patients suffered from LGM in Week 6 of CRT (end of study).

**FIGURE 4:**
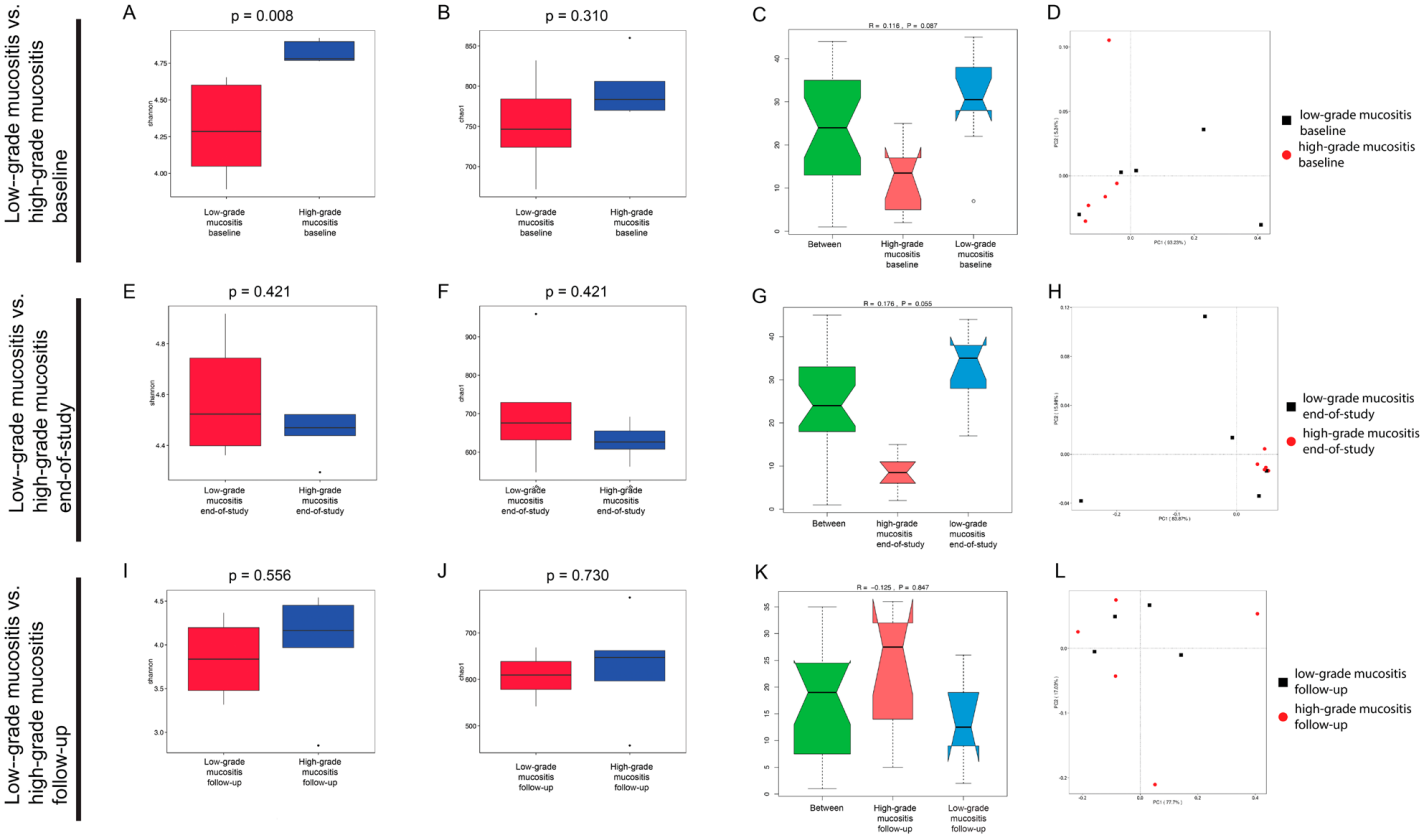
Evaluation of differences between patients with low‐grade mucositis vs high‐grade mucositis over time. (A, B, E, F, I, J) Comparison of alpha‐diversity indices (Shannon and Chao1) of patient subgroups. Upper panels: patients with low‐grade mucositis vs. high‐grade mucositis at baseline. patients with low‐grade mucositis vs. high‐grade mucositis at the end of the study. Lower panels: patients with low‐grade mucositis vs. high‐grade mucositis during follow‐up. (C, G, K) ANOSIM analysis comparing low‐grade mucositis and high‐grade mucositis patients’ samples at different time points with respect to their similarity at the phylum level. The green boxplot shows the inter‐group variance, while the red boxplots show the intra‐group variance of the high‐grade mucositis subgroup and the blue boxplot shows the intra‐group variance of the low‐grade mucositis subgroup. (D, H, L) Principal coordinate analysis (PCoA) plot based on Bray‐Curtis distance visualizing the overall structure of saliva microbiota of low‐grade mucositis and high‐grade mucositis patients at different time points.

At baseline, the Shannon index on the genus level was statistically significantly higher in the HGM group than that in the LGM group (*p* = 0.008) (Figure 4A). The Chao 1 index did not show significant differences between the HGM and LGM groups at baseline (Figure 4B). At the end of the study and during follow‐up, alpha diversity indices did not show significant differences between the HGM and LGM groups (Figure 4E,F,I,J).

The beta diversity showed no significant intra‐ or intergroup variance between HGM and LGM at any time point (Figure 4C,G,K), although we found a higher intergroup variance during CRT Week 6 (end of study), which was not statistically significant (*p* = 0.055) (Figure 4G).

At baseline and during follow‐up, all samples showed a broad overlap with no clear subclustering (Figure 4D,L). Bray–Curtis–based PCoA revealed a stronger clustering of HGM patients at the end of the study than that of LGM patients (Figure 4H).

**FIGURE 5:**
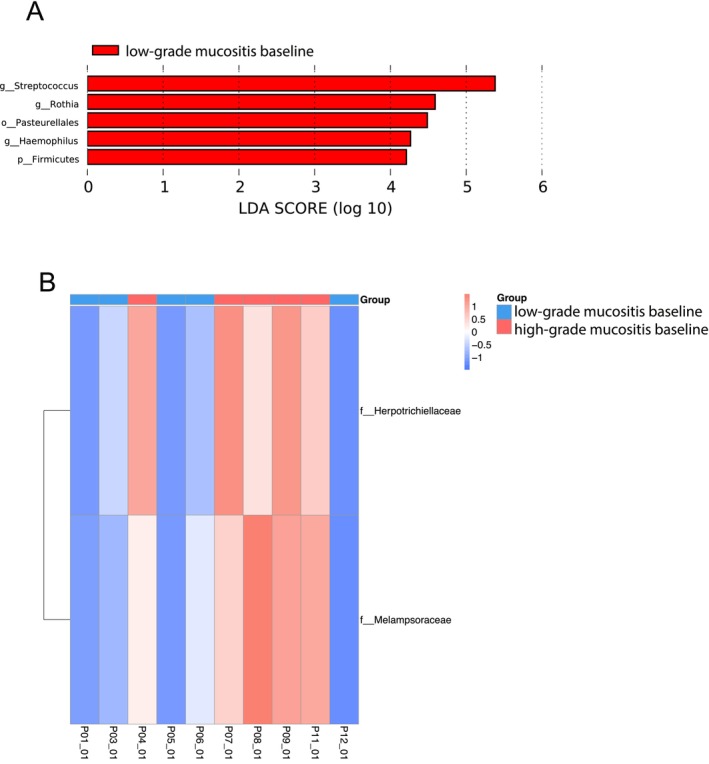
(A) Linear discriminant analysis effect size (LEfSe)‐based analysis showing differentially abundant microbial taxa between low‐grade mucositis and high‐grade mucositis subgroups at baseline. (B) Taxonomic abundance heat map based on LEfSe of low‐grade mucositis and high‐grade mucositis subgroups at baseline. The color code ranges from blue (low abundance) to red (high abundance).

At the end of the study, no microbial taxa were found to be differentially abundant between the HGM and LGM subgroups. Yet, we found an enrichment of the *Rothia* genus, the Firmicutes phylum including *Streptococcus* spp., and the Pasteurellales order including *Haemophilus* spp. in the LGM group at baseline (Figure 5A). Additionally, we found two fungal families to be significantly more abundant in the HGM group at baseline, namely, Melampsoraceae and Herpotrichiellaceae (Figure 5B).

### 3.4 Changes in the Oral Microbiota of Healthy Controls Over Time

We did not observe significant differences in alpha or beta diversity in healthy controls over time (Figure S5A–C). Also, no differentially abundant taxa were found. Samples belonging to the same healthy individual typically clustered together over time, that is, were closer to each other than to samples of different individuals at the same time points (Figure S5D). Active smokers clustered at the edges of the dendrogram. Subsequently, we divided the control group into two subgroups according to their nicotine consumption (active smoking [*n* = 3] vs. never smoked [*n* = 4]) to compare their oral microbial community structure. Our analysis revealed the Proteobacteria phylum, including the Neisseriales order and *Neisseria* genus and the Pasteurellales order, including the *Haemophilus* genus, to be related to nonsmokers, whereas the Actinomycetaceae family was related to active smokers. Additionally, *Oscillibacter valericigenes* species was found to be significantly enriched among active smokers (Figure S6D).

## 4 Discussion

Taxa found to be enriched in controls in our study at any time are mostly known to be commensals of the oral cavity and ubiquitous members of the human oral microbiome [24]. *Streptococcus*, *Actinomyces*, *Rothia*, *Neisseria*, and *Veillonella* belong to the five most abundant genera in our study in patients during CRT and controls. *Streptococcus* spp., such as *S. salivarius* , *S. timonensis*, and *S. australis* as well as various *Actinomyces* spp., were enriched in controls. These findings correlate with other studies, where the total number of streptococci, including *S. sanguis*, *S. oralis* , and *S. salivarius*, was associated with good oral health [10]. *Actinomyces* spp. were also associated with the healthy oral microbiome [24].

Furthermore, we showed that the oral microbiome of controls remained stable when viewed longitudinally over 3 months, as previously postulated by Costello et al. [25]. Using 16S rRNA gene sequencing, Costello et al. showed that different body sites, including the oral cavity, harbored a relatively stable set of characteristic microbiota across people and over time in the absence of external influences [25]. By implementing a prospective longitudinal design instead of a cross‐sectional design, we were able to compare individuals over time, thereby minimizing the effects of interindividual variation. Furthermore, adding a control group helped with the robustness of our approach.

In our study, many species had a significantly decreased abundance in patients undergoing CRT. These findings show that CRT had a substantial impact on the salivary microbial profile of HNSCC patients, which is congruent with previous studies [26, 27]. In Week 6 of CRT, we detected a less diverse oral microbiome compared to the oral microbiome at baseline, whereas evenness did not differ significantly. End‐of‐study samples had a higher microbial composition similarity than non‐irradiated samples (baseline) and vice versa. We showed that with increased cumulative radiation dose and increased severity of mucositis (mid‐study vs. end of study), the changes in the oral microbiome became more and more distinguishable.

Interestingly, only two species were found to have a significantly higher relative abundance in patients at the end of study compared to patients at baseline, namely, *S. aureus* and *E. coli* . *S. aureus* and *E. coli* are facultative anaerobes and have been isolated from the saliva of oral cancer patients undergoing CRT in previous studies [28]. It was shown that there is an increased prevalence of *S. aureus* in the ulcerative phase of RIOM following CRT and that *Staphylococcus* spp. was associated with the presence of ulcerative oral mucositis [27]. This opportunistic bacterium is commonly associated with nonoral infections and diseases but also oral superinfections and episodes of bacteremia in patients with oral mucositis [29]. Bacteremia following oral mucositis is most frequently associated with aerobic Gram‐positive cocci (in particular, coagulase‐negative *Staphylococcus* spp., *Streptococcus viridans*, or *S. aureus* ) or aerobic Gram‐negative bacilli (especially *E. coli* , *Klebsiella pneumoniae* , or *Pseudomonas aeruginosa* ) [9]. Sonalika et al. found *E. coli* , among other species, to possibly contribute to exacerbation of mucositis [30]. Combined, our findings suggest that there is a dysbiosis of the oral microbiota among patients undergoing CRT and a shift in the microbiota profile to potentially more pathogenic taxa. Gaining a deeper understanding of the role of oral microbiota in the pathobiology of oral mucositis is key to developing preventive and treatment strategies for local or systemic and possibly life‐threatening infections. In a randomized, double‐blind study, Sharma et al. showed that *Lactobacillus brevis* CD2 lozenges reduced the incidence of grade III and IV anticancer therapy‐induced oral mucositis due to its potential anti‐inflammatory activity in patients undergoing hematopoietic stem cell transplantation [31]. We did not test any pre‐ or probiotic treatments in our patients. To date, no approved pre‐ or probiotic strategy has achieved sufficient evidence to become standard of care. Additional studies are needed to provide a deeper functional analysis by using metabolomics, meta‐proteomics, and meta‐transcriptomics to assess the causality of the shifts in the oral microbiome during CRT and to eventually detect microbes with therapeutic potential.

During CRT, our analysis detected 36 taxa to be enriched in controls compared to patients versus 12 taxa already found to be enriched in controls compared to patients at baseline. Additionally, 35 microbial species were revealed with significant differences in relative abundance between patients during CRT and healthy controls. Furthermore, beta diversity comparing patients and controls only showed a significant intergroup variance at the phylum level during CRT and not at the other time points. These findings show once more that the oral microbiome changes significantly during cancer treatment.

Differentially abundant taxa in controls versus patients at baseline, including *Rothia* spp. and *Sanguibacter* spp., could indicate links between the oral microbiome and HNSCC. However, it should be taken into consideration that there has been a heterogeneity between patients and controls according to baseline criteria, including gender and age. It is likely that these patient‐related factors may have contributed to the differences in the oral microbiome at baseline. During follow‐up, the differential abundance of taxa between patients and controls observed during CRT could not be detected anymore, showing that changes in bacterial composition caused by CRT appear to be transient.

One taxon, namely, the Leuconostocaceae family of the Lactobacillales order, had a significantly higher relative abundance in patients at baseline. Thus, it could be of importance to further investigate its role regarding the pathogenesis of HNSCC. Previously, Sonalika et al. showed that patients with OSCC (“oral squamous cell carcinoma”) harbored a significantly higher number of oral aerobes, anaerobes, coliforms, and Gram‐negative anaerobic bacteria compared to healthy individuals [30]. We found that taxa shown to be associated with human infections were enriched in patients undergoing CRT [9]. NGS allows the analysis of microorganisms that are not amenable to cultivation, although, due to different methodological approaches or different study populations, results are not always directly comparable. Almstahl et al. reported the association of cultivable *Candida albicans* with radiation‐induced hyposalivation and the presence of *Lactobacillus* spp. in over 90% of RT patients [10]. Two studies assessing the oral bacterial profiles using 16S rRNA gene sequencing with different study populations than ours—active disease nasopharyngeal carcinoma patients, all receiving radiotherapy, with or without additional chemotherapy [26], and multiple myeloma patients treated with high‐dose melphalan followed by autologous SCT (auto‐SCT) [27]—observed a significant decrease in microbial diversity after the cancer treatment. We could confirm these findings.

Of note, we identified a potential association of the *Capnocytophaga* genus with patients receiving dCRT without tumor operation versus aCRT, which is another indicator that HNSCC tissue itself influences the composition of the oral microbiome. The *Capnocytophaga* genus is considered to be OSCC related [32, 33]. Mager et al. showed that *Capnocytophaga gingivalis* , as well as *Prevotella melaninogenica* and *Streptococcus mitis* , counts were significantly increased among OSCC patients compared to cancer‐free subjects and that these species could even be used as diagnostic markers for oral cancer with a relatively high sensitivity and specificity (both > 80%) [34]. Zhu et al. suggested that *C. gingivalis* might invade OSCC tissues and play an important role in carcinogenesis by promoting OSCC invasion and metastasis by inducing epithelial‐to‐mesenchymal transition (EMT) [35].

Furthermore, we investigated potential differences between LGM and HGM subgroups with respect to the microbial profile. In the ulcerative phase of RIOM, microorganisms growing in a biofilm show much greater resistance to antimicrobial agents than their free‐living microbiota [26]. This further challenges the effective treatment of severe mucositis. Owing to pronounced pain, dysphagia, and xerostomia, severe RIOM causes a significant decrease in quality of life and may even lead to discontinuation of oncological treatment [36]. At baseline, the HGM group harbored more diverse and more even oropharyngeal taxa than the LGM group as a significantly higher Shannon index accounts for both richness and evenness of the taxa present, whereas the Chao 1 index only measures richness. During CRT, the HGM subgroup had a higher similarity of microbial composition than the LGM subgroup. There was a higher intergroup variance of LGM versus HGM during irradiation, although this separation was not significant, which could be due to the power of the study.

Interestingly, in our study, patients who developed HGM exhibited some distinct microbial taxa at baseline that differentiated them from patients who did not develop HGM, namely, two fungal families, Melampsoraceae and Herpotrichiellaceae. Melampsoraceae may be associated with the environment rather than the human microbiome [37]. Also, Herpotrichiellaceae has not yet been linked to RIOM or HNSCC, but there have been opportunistic, human‐pathogenic species reported in the Herpotrichiellacea family [38]. To be able to distinguish microbial taxa that could serve as potential biomarkers to identify patients who are at risk of developing HGM, further investigations are needed. Taxa associated with the LGM group at baseline, at the genus level, for example, *Rothia* spp., *Streptococcus* spp., and *Haemophilus* spp., are known commensals of the oral microbiome [24].

Unexpectedly, no microbial taxa were found to be statistically significantly distinct between the LGM and HGM groups at the end of the study. The differentially abundant taxa found between LGM and HGM at baseline were not found to be differentially abundant anymore during irradiation. These findings are in contrast to previous data where Zhu et al. showed that from the occurrence of visible erythema to the beginning of severe mucositis (RTOG 3), patients in the mild subgroup harbored significantly more diverse bacteria and that patients who eventually developed severe RIOM transiently harbored a notably higher proportion of *Actinobacillus* spp. during a mild phase of RIOM. They postulated that *Actinobacillus* spp. may profoundly affect oropharyngeal microbial homeostasis and be one of the associated factors that predispose patients to severe mucositis. We could not reproduce these findings. Furthermore, we did not find an association between *Candida* spp. and RIOM as reported in previous studies [10]. However, this may be due to inherent differences between the studies, including, among others, differences in treatment modalities, cancer sites, or sequencing methods.

Notably, our data did not indicate a significant impact of antibiotic treatment on the microbial changes triggered by CRT. This might be due to the resilience of the oral microbiome, as investigated in other studies. It has been shown that antibiotics have a minor and only transient effect on the oral microbiome compared to the gut microbiome [39]. Zaura et al. concluded that healthy individuals, exposed to a single antibiotic treatment, undergo considerable microbial shifts and enrichment in antibiotic resistance in their feces, whereas their salivary microbiome composition remains stable [39]. However, ABT is not expected to affect all microbial organisms; for example, fungal and viral genes are excluded.

In our functional analysis, the ARGs with the highest relative percentage in the patient's group during CRT were CTX‐M‐6, gadX, and cat. CTX‐M‐6 belongs to the structural family of extended‐spectrum β‐lactamases. Initially, CTX‐M‐type beta‐lactamases were reported in the 1980s. Since then, their rate of dissemination among bacteria has increased rapidly [40]. They are encoded by transferable plasmids and found in various enterobacteria. CTX‐M‐6, for example, is found in *Salmonella typhimurium* [41]. GadX is an AraC family regulator and plays an important role in multidrug resistance [42], whereas the major mechanism of resistance to chloramphenicol is the production of the chloramphenicol acetyltransferase (cat) gene [43]. Overall, we could see that the ARGs profile was more diverse in the control group than in the patient cohort, which could be due to the effects of CRT. Functional analysis of microbiomes not only allows the detection of ARGs but helps to get a deeper understanding of the microbial profile by exploring gene families, metabolic pathways, and systems to identify the functional capabilities of the detected taxa [44]. Providing a detailed functional analysis was beyond the scope of our pilot study but should be explored more in future studies.

Two limitations of our study were the sample size and the heterogeneity between patients and controls according to baseline criteria. We assessed additional parameters, such as oral hygiene and HPV status, as HPV positivity has been established as a favorable prognostic factor in oropharyngeal carcinoma, but we did not correlate these parameters with the changes in the patients' oral microbiome. Therefore, we cannot comment on the role of HPV status in the development of (high‐grade) oral mucositis. However, analyzing the potential role of HPV status in the development of high‐grade oral mucositis could be an interesting approach and should be considered in future studies. Notably, our patient cohort was very homogenous in terms of treatment modalities, radiation dose, cancer sites, and demographics. The sampling site as such may also influence the results, although it has been shown that saliva samples are representative oral samples for microbiome studies in HNSCC patients [45]. As many of our patients suffered from hyposalivation mostly at the end of study and during follow‐up, this circumstance could also have influenced our results.

In conclusion, we assessed the dynamics of the relationship between the oral mucositis of HNSCC patients and controls before, during, and up to 9 months after CRT, and showed that the oral microbiome differed between patients and controls before irradiation started. We demonstrated that CRT of HNSCC patients induced significant, although transient, changes in the oral microbiome and that these changes correlated with the progression and aggravation of RIOM. Interestingly, patients who developed HGM exhibited microbial taxa at baseline were distinct from patients who did not develop HGM. During irradiation, no differentially abundant taxa were found between the LGM and the HGM subgroups. Furthermore, *Capnocytophaga* spp., a genus considered to be OSCC related, was enriched in patients treated with dCRT versus those treated with aCRT. Hence, our study serves as a pilot for further research on larger cohorts to gain a deeper understanding of the impact of the oral microbiome in health and disease and to identify potential biomarkers to screen patients who are at risk of developing HGM.

## Author Contributions

**Dominique A. Torozan:** conceptualization (equal), investigation (equal), project administration (equal), validation (equal), visualization (equal), writing – original draft (lead). **Cédric Christian Laczny:** conceptualization (equal), data curation (equal), methodology (equal), validation (supporting), writing – review and editing (supporting). **Kirsten Roomp:** writing – review and editing (equal). **Paul Wilmes:** methodology (equal), writing – review and editing (equal). **Jochen Fleckenstein:** conceptualization (lead), funding acquisition (supporting), investigation (lead), methodology (lead), project administration (lead), resources (supporting), supervision (lead), writing – original draft (supporting), writing – review and editing (supporting). **Jochen G. Schneider:** conceptualization (lead), funding acquisition (lead), investigation (lead), project administration (lead), resources (lead), supervision (lead), writing – original draft (supporting), writing – review and editing (lead).

## Ethics Statement

The study design was approved by the local ethics committee (EK‐Nr. 291/16). A written informed consent form was signed by each participant prior to inclusion.

## Consent

All authors agreed on the submission in the current format.

## Conflicts of Interest

The authors declare no conflicts of interest.

## Supporting information


**Figure S1.** Comparison of relative taxonomic abundances of patient samples and control samples mid‐study. Taxonomic profiles of the microbial communities at (A) the phylum level and (B) the genus level. The top 10 most abundant taxa in terms of relative abundance are shown. All other taxa are grouped under “Others”.
**Figure S2.** (A–C) Linear discriminant analysis effect size (LEfSe)‐based analysis identifying differentially abundant microbial taxa between patients and controls at different time points. Only taxa meeting an LDA threshold > 4 are shown. c, class; f, family; g, genus; o, order; P, phylum; s, species.
**Figure S3.** (A, B, D, E, G, H) Comparison of alpha‐diversity indices (Shannon and Chao1) of patient subgroups. Upper panels: patients with definitive chemoradiotherapy (dCRT) versus patients with adjuvant chemoradiotherapy (aCRT). Middle panels: patients at baseline versus patients at the end of study. Lower panels: patients before antibiotic (ABX) administration versus patients during ABX administration. In all box plots: box hinges, first and third quartiles; whiskers, hinge to highest/lowest values that are within 1.5 × IQR of hinge. (C, F, I) ANOSIM analysis comparing patient subgroups with respect to their similarity at the phylum level. The green box plot shows the intergroup variance, whereas the red and blue box plots show the intragroup variance of the different groups.
**Figure S4.** Linear discriminant analysis effect size (LEfSe) identifying differentially abundant microbial taxa between (A) patients treated with dCRT versus aCRT at baseline and (B) patients at baseline versus end of the study (CRT week 6). Only taxa meeting an LDA threshold > 4 are shown. c, class; f, family; g, genus; o, order; P, phylum; s, species.
**Figure S5.** Evaluation of differences between controls over time. (A, B) Comparison of alpha‐diversity indices (Shannon and Chao1) of control subgroups. In all box plots: box hinges, first and third quartiles; whiskers, hinge to highest/lowest values that are within 1.5 × IQR of hinge. (C) ANOSIM analysis comparing controls at baseline versus at the end of the study with respect to their similarity at the phylum level. The green box plot shows the intergroup variance, whereas the red and blue box plots show the intragroup variance of the different groups. (D) Sample clustering analysis based on Bray–Curtis distance of the relative taxonomic abundance on the phylum level of controls over time.
**Figure S6.** (A, B) Box plots of alpha‐diversity indices (Shannon and Chao1) comparing nonsmokers versus smokers in the control group. (C) Box plots of ANOSIM analysis testing statistically significant differences between nonsmokers and smokers in the control group. The green box plot shows the intergroup variance, whereas the red and blue box plots show the intragroup variance of the different groups. (D) Microbial taxon with a significantly different relative abundance (*q* < 0.05). (E) Linear discriminant analysis effect size (LEfSe) identifying the most differentially abundant microbial taxa between nonsmokers and smokers in the control group. Only taxa meeting an LDA threshold > 4 are shown. c, class; f, family; g, genus; o, order; P, phylum; s, species. In all box plots: box hinges, first and third quartiles; whiskers, hinge to highest/lowest values that are within 1.5 × IQR of hinge.
**Table S1.** CONSORT (Consolidated Standards of Reporting Trials) Flowchart. CRT, chemoradiotherapy; HNSCC, head and neck squamous cell cancer.

## Data Availability

Raw sequencing files obtained in this study can be uploaded and made available by request.
