# Supplementary material for: Metagenomic Profiling of Oral Microbiome Dynamics During Chemoradiotherapy in Head and Neck Squamous Cell Carcinoma Patients
Source: Cancer Med. 2025 Jan 13;14(1):e70589. doi: 10.1002/cam4.70589 (PMC11725981; doi:10.1002/cam4.70589)
Supplement: Supplementary file 1 — Figure S1. Comparison of relative taxonomic abundances of patient samples and control samples mid‐study. Taxonomic profiles of the microbial communities at (A) the phylum level and (B) the genus level. The top 10 most abundant taxa in terms of relative abundance are shown. All other taxa are grouped under “Others”. Figure S2. (A–C) Linear discriminant analysis effect size (LEfSe)‐based analysis identifying differentially abundant microbial taxa between patients and controls at different time points. Only taxa meeting an LDA threshold > 4 are shown. c, class; f, family; g, genus; o, order; P, phylum; s, species. Figure S3. (A, B, D, E, G, H) Comparison of alpha‐diversity indices (Shannon and Chao1) of patient subgroups. Upper panels: patients with definitive chemoradiotherapy (dCRT) versus patients with adjuvant chemoradiotherapy (aCRT). Middle panels: patients at baseline versus patients at the end of study. Lower panels: patients before antibiotic (ABX) administration versus patients during ABX administration. In all box plots: box hinges, first and third quartiles; whiskers, hinge to highest/lowest values that are within 1.5 × IQR of hinge. (C, F, I) ANOSIM analysis comparing patient subgroups with respect to their similarity at the phylum level. The green box plot shows the intergroup variance, whereas the red and blue box plots show the intragroup variance of the different groups. Figure S4. Linear discriminant analysis effect size (LEfSe) identifying differentially abundant microbial taxa between (A) patients treated with dCRT versus aCRT at baseline and (B) patients at baseline versus end of the study (CRT week 6). Only taxa meeting an LDA threshold > 4 are shown. c, class; f, family; g, genus; o, order; P, phylum; s, species. Figure S5. Evaluation of differences between controls over time. (A, B) Comparison of alpha‐diversity indices (Shannon and Chao1) of control subgroups. In all box plots: box hinges, first and third quartiles; whiskers, hinge to highest/l [file CAM4-14-e70589-s001.docx]

**Supplement Figure 1.** Comparison of relative taxonomic abundances of patient samples and control samples mid-study. Taxonomic profiles of the microbial communities at **(A)** the phylum level and **(B)** the genus level. The top 10 most abundant taxa in terms of relative abundance are shown. All other taxa are grouped under “Others”.

**
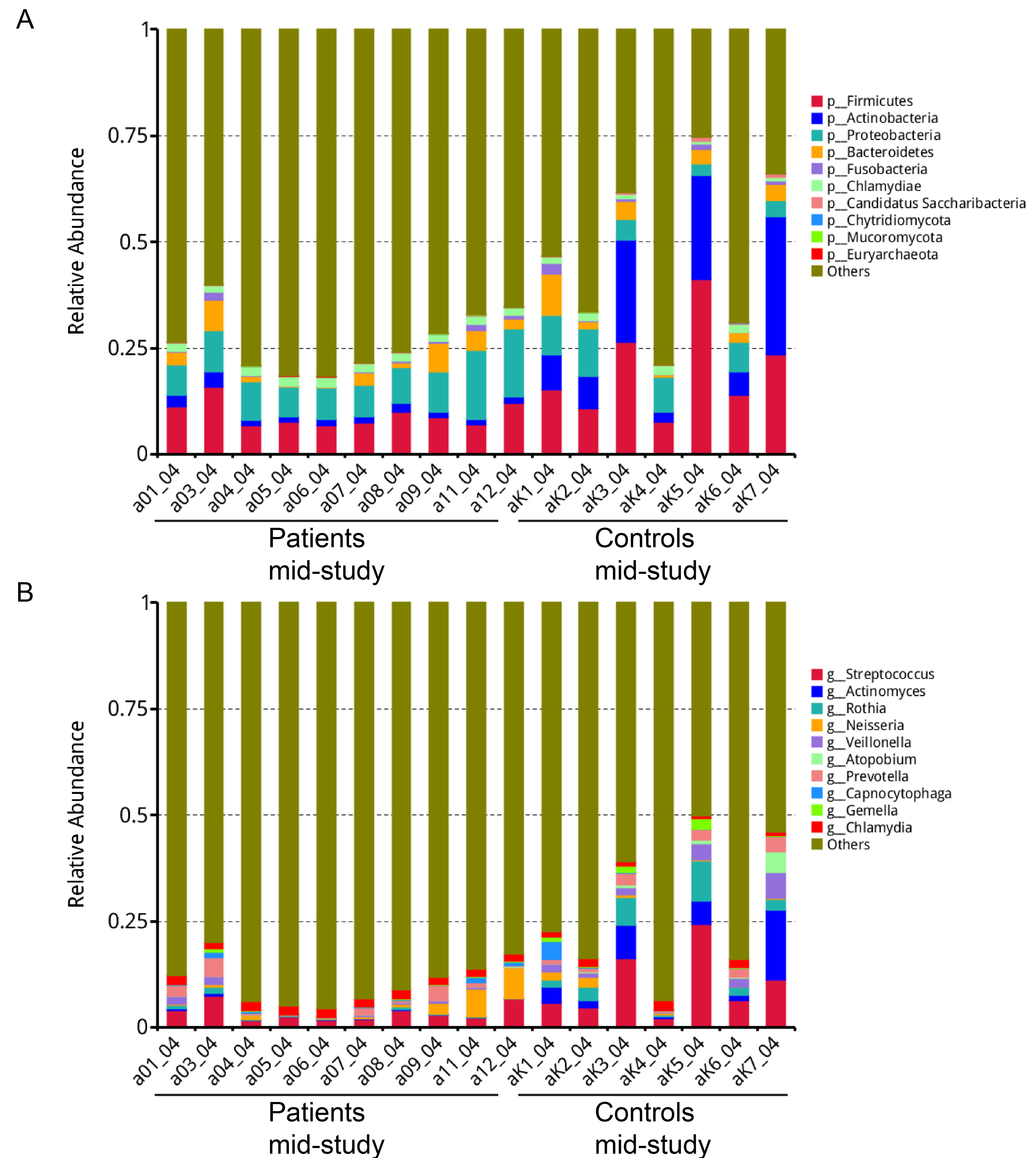
**

**Supplement Figure 2. (A/B/C)** Linear Discriminant Analysis Effect Size (LEfSe)-based analysis identifying differentially abundant microbial taxa between patients and controls at different time points. Only taxa meeting an LDA threshold > 4 are shown. P- phylum; c- class; o- order; f- family, g- genus; s- species.


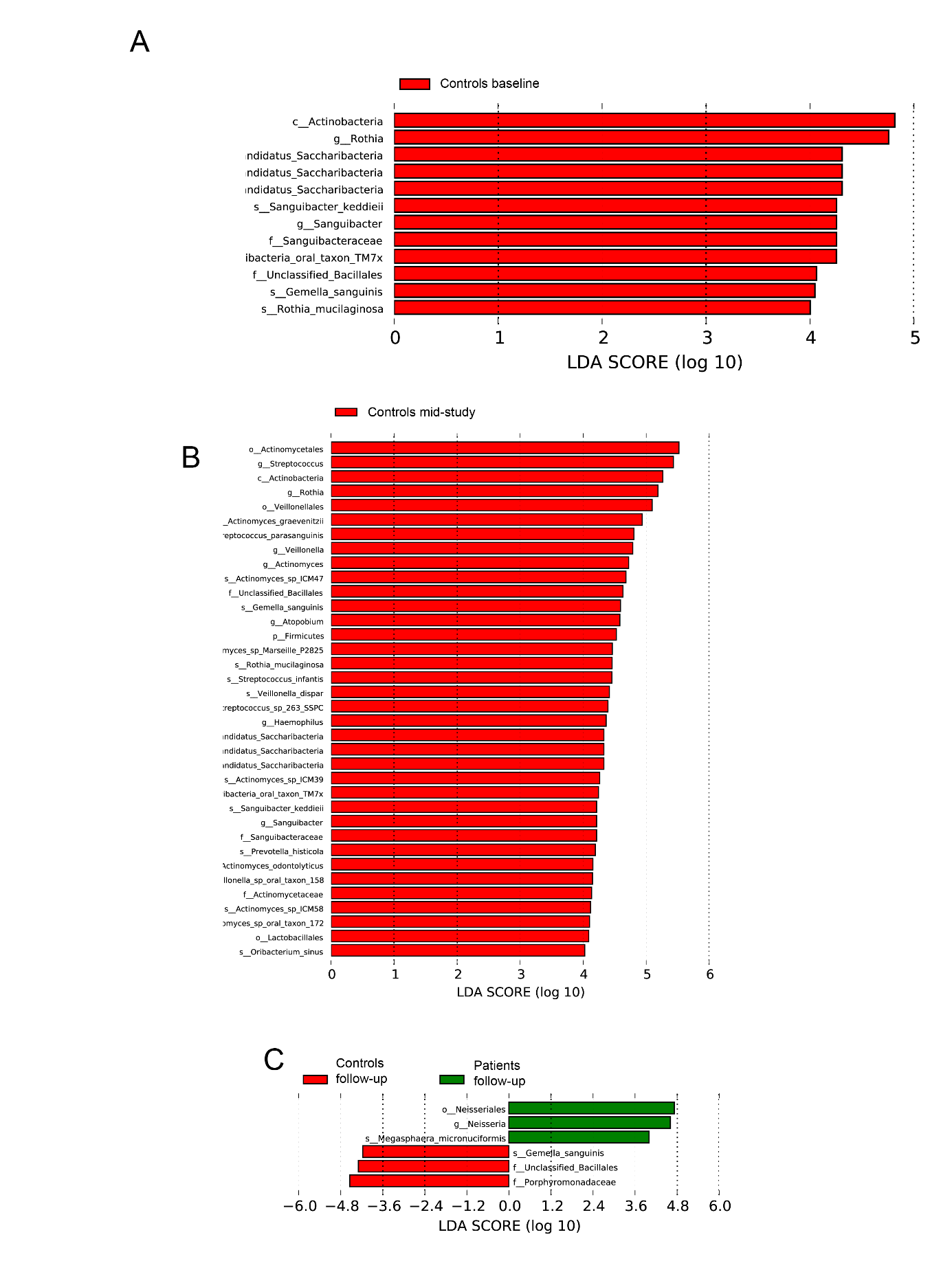


**Supplement Figure 3. (A/B/D/E/G/H)** Comparison of alpha-diversity indices (Shannon and Chao1) of patient subgroups. Upper panels: patients with definitive CRT (dCRT) vs. patients with adjuvant CRT (aCRT). Middle panels: patients at baseline vs. patients at end of study. Lower panels: patients before antibiotic (ABX) administration vs. patients during ABX administration. In all box plots: box hinges: 1st and 3rd quartiles; whiskers: hinge to highest/lowest values that are within 1.5*IQR of hinge. (C/F/I) ANOSIM analysis comparing patient subgroups with respect to their similarity at the phylum level. The green boxplot shows the inter-group variance, while the red and blue boxplots show the intra-group variance of the different groups.

**
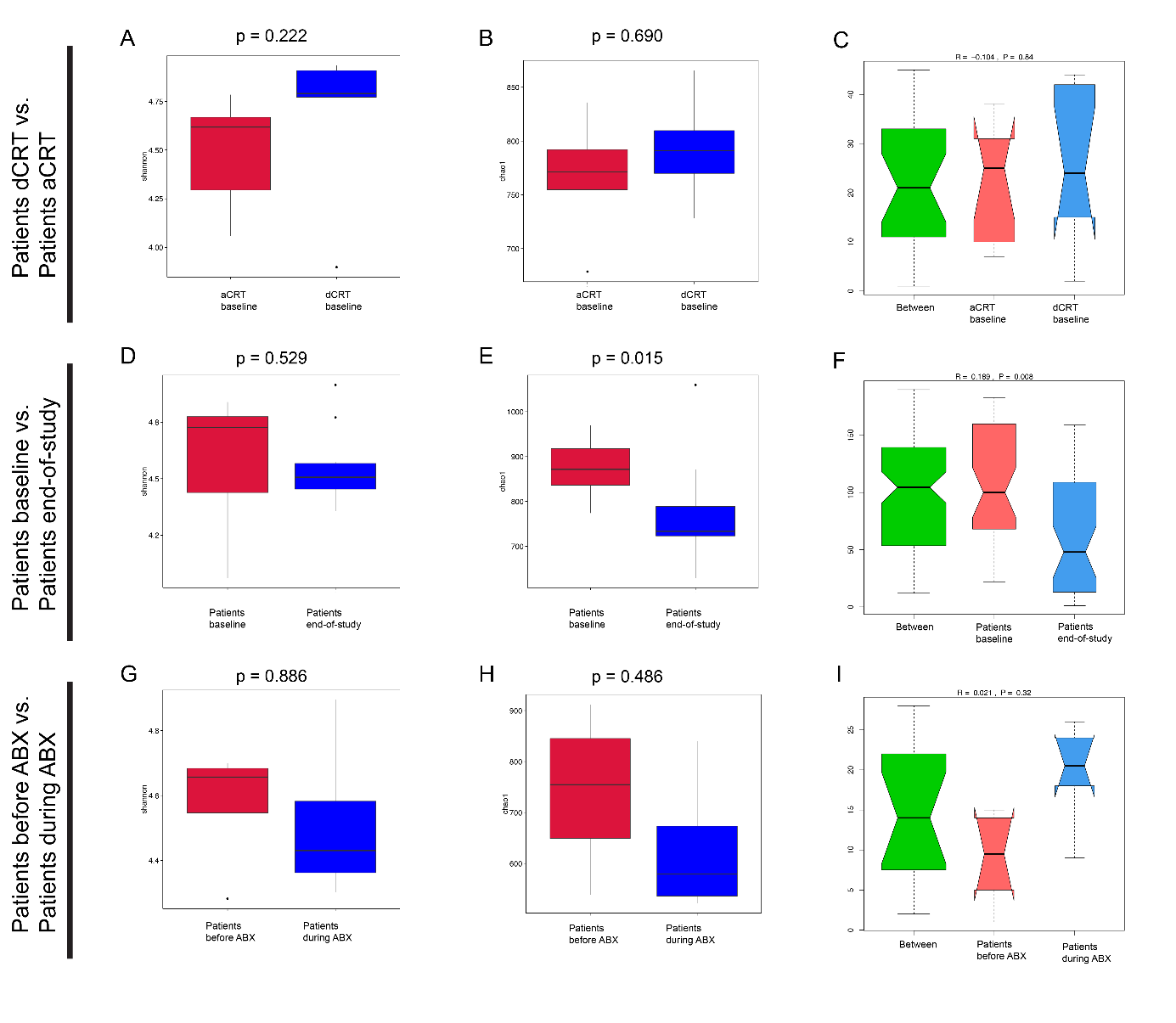
**

**Supplement Figure 4.** Linear Discriminant Analysis Effect Size (LEfSe) identifying differentially abundant microbial taxa between **(A)** patients treated with dCRT vs. aCRT at baseline and **(B)** patients at baseline vs. end-of-study (CRT week 6). Only taxa meeting an LDA threshold > 4 are shown. P- phylum; c- class; o- order; f- family, g- genus; s- species.

**
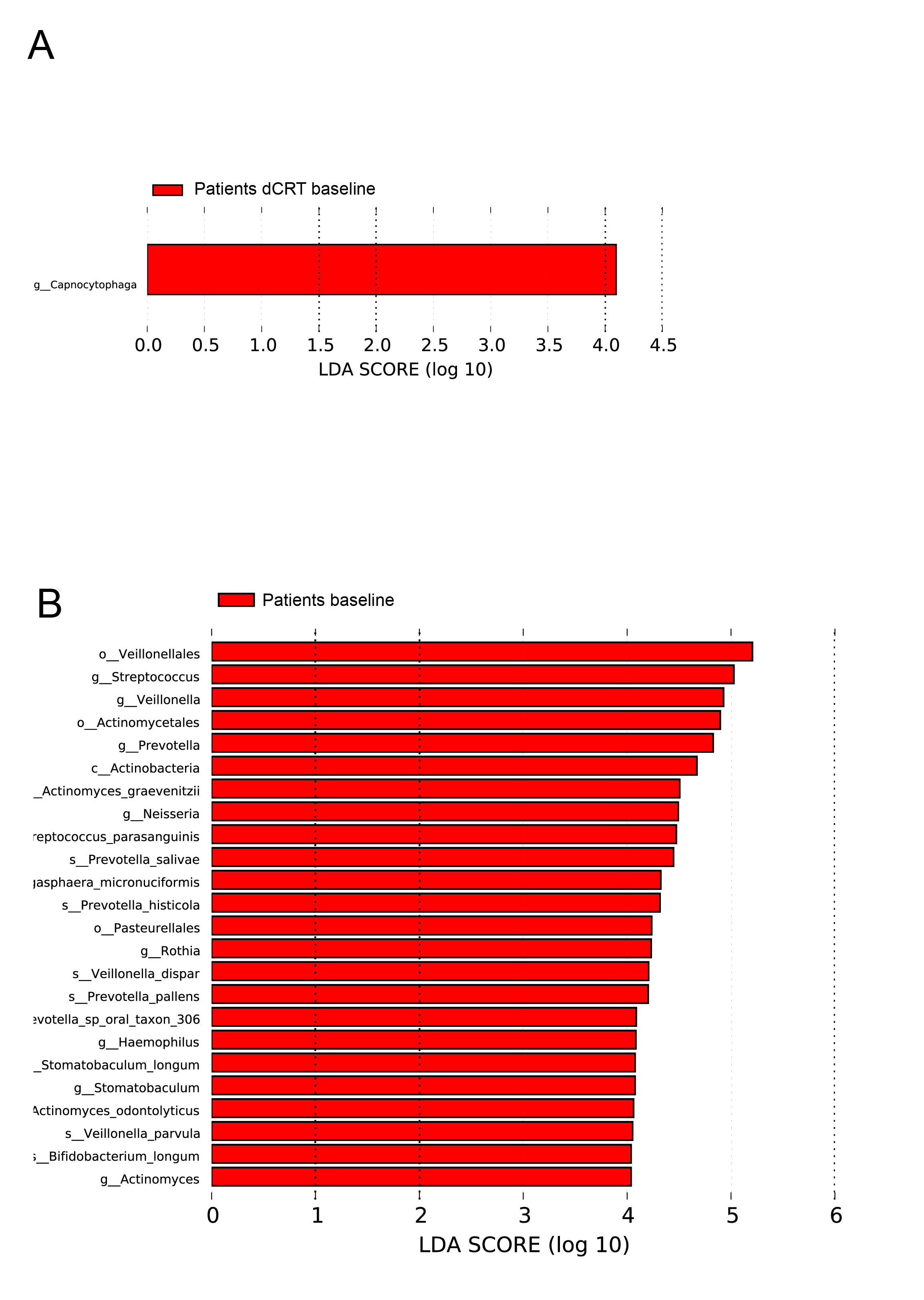
**

**Supplement Figure 5.** Evaluation of differences between controls over time. **(A/B)** Comparison of alpha-diversity indices (Shannon and Chao1) of control subgroups. In all box plots: box hinges: 1st and 3rd quartiles; whiskers: hinge to highest/lowest values that are within 1.5*IQR of hinge. (C) ANOSIM analysis comparing controls at baseline vs. at the end of the study with respect to their similarity at the phylum level. The green boxplot shows the inter-group variance, while the red and the blue boxplot show the intra-group variance of the different groups. (**D)** Sample clustering analysis based on Bray-Curtis distance of the relative taxonomic abundance on the phylum level of controls over time.


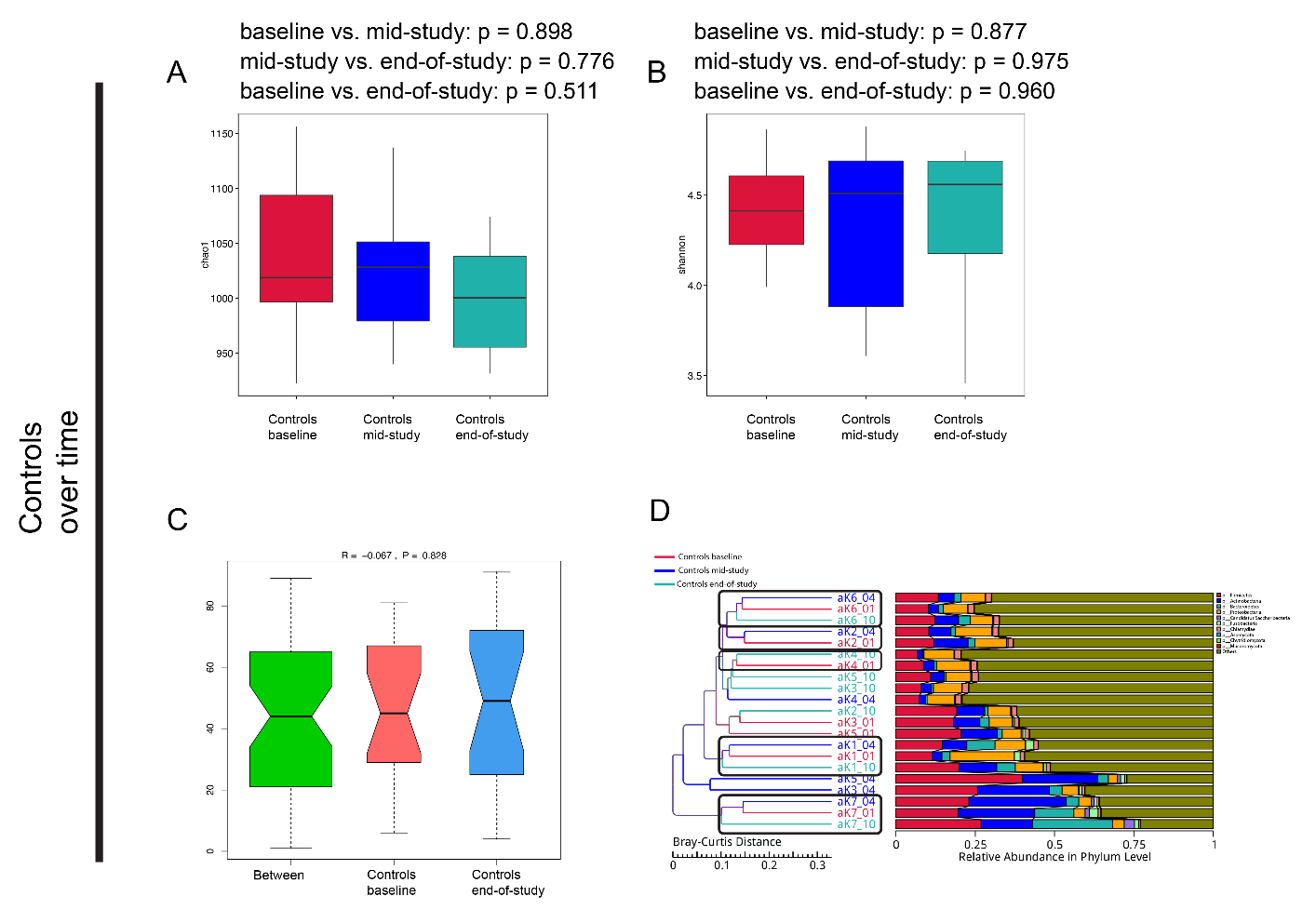


**Supplement Figure 6. (A/B)** Box plots of alpha-diversity indices (Shannon and Chao1) comparing non-smokers vs. smokers in the control group. **(C)** Box plots of ANOSIM analysis testing statistically significant differences between non-smokers and smokers in the control group. **(D)** Microbial taxon with significantly different relative abundance (q < 0.05). **(E)** Linear Discriminant Analysis Effect Size (LEfSe) identifying the most differentially abundant microbial taxa between non-smokers and smokers in the control group. Only taxa meeting an LDA threshold > 4 are shown. P- phylum; c- class; o- order; f- family, g- genus; s- species. In all box plots: box hinges: 1st and 3rd quartiles; whiskers: hinge to highest/lowest values that are within 1.5*IQR of hinge.


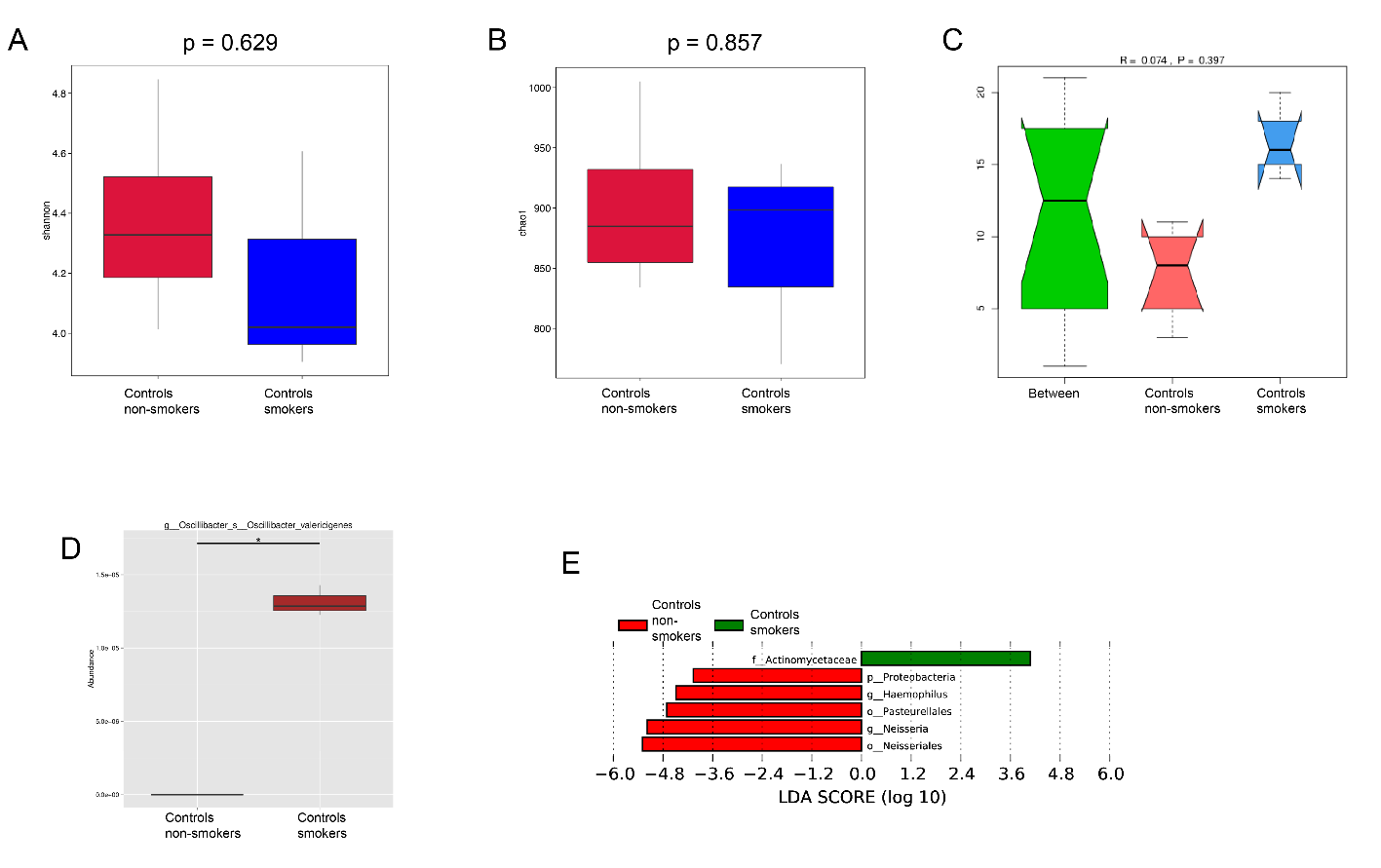


**Supplement Table 1.** CONSORT (Consolidated Standards of Reporting Trials) Flowchart. Abbreviations: HNSCC = head and neck squamous cell cancer, CRT = chemoradiotherapy
